# A comprehensive next generation sequencing tissue assay for Asian-prevalent cancers—Analytical validation and performance evaluation with clinical samples

**DOI:** 10.3389/fmolb.2022.963243

**Published:** 2022-09-21

**Authors:** Cedric Chuan-Young Ng, Sandy Lim, Abner Herbert Lim, Nur Diyana Md Nasir, Jingxian Zhang, Vikneswari Rajasegaran, Jing Yi Lee, Jessica Sook Ting Kok, Aye Aye Thike, Johnathan Xiande Lim, Ruifen Weng, Sidney Yee, Yukti Choudhury, Jason Yongsheng Chan, Puay Hoon Tan, Min-Han Tan, Bin Tean Teh

**Affiliations:** ^1^ Cancer Discovery Hub, National Cancer Centre Singapore, Singapore, Singapore; ^2^ Laboratory of Cancer Epigenome, National Cancer Centre Singapore, Singapore, Singapore; ^3^ Diagnostics Development Hub (DxD Hub), A National Platform Hosted by A*STAR, Singapore, Singapore; ^4^ Department of Anatomical Pathology, Singapore General Hospital, Singapore, Singapore; ^5^ Division of Pathology, Singapore General Hospital, Singapore, Singapore; ^6^ Lucence Diagnostics Pte Ltd, Singapore, Singapore; ^7^ Division of Medical Oncology, National Cancer Centre Singapore, Singapore, Singapore

**Keywords:** next-generation sequence (NGS), hybrid capture, molecular diagnostics, asian cancers, genetic alteration, tissue assay

## Abstract

**Introduction:** A well-validated diagnostic assay with curated biomarkers complements clinicopathological factors to facilitate early diagnosis and ensure timely treatment delivery. This study focuses on an Asian-centric cancer diagnostic assay designed and thoroughly validated against commercially available standard references and a cohort of over 200 clinical specimens spanning 12 diverse Asian-centric cancer types.

**Methods:** The assay uses hybrid-capture probes capable of profiling DNA aberrations from 572 cancer-related genes and 91 RNA fusion partners. The panel can detect clinically-tractable biomarkers such as microsatellite instability (MSI) and tumor mutation burden (TMB).

**Results:** Analytical evaluation demonstrated 100% specificity and 99.9% sensitivity within a ≥5% VAF limit of detection (LoD) for SNV/Indels. RNA-based fusion features an LoD of ≥5 copies per nanogram input when evaluated against commercial references. Excellent linearity and concordance were observed when benchmarking against orthogonal methods in identifying MSI status, TMB scores and RNA fusions. Actionable genetic alterations were identified in 65% of the clinical samples.

**Conclusion:** These results demonstrate a molecular diagnostic assay that accurately detects genomic alterations and complex biomarkers. The data also supports an excellent performance of this assay for making critical diagnoses and well-informed therapeutic decisions in Asian prevalent cancers.

## 1 Introduction

A recent survey conducted by GLOBOCAN estimates cancer development in at least one in five individuals during their lifetime. Across the globe, Asians contribute to 60% of the world’s cancer burden, and in 2020 alone, GLOBOCAN reported 5.8 million cancer-related deaths (Sung et al., 2021). As the knowledge between pathophysiology and genetic variation begins to expand, high-throughput molecular diagnostic assays have increased the deployment of next-generation sequencing (NGS) technologies in the clinical practice of diagnostic (Meldrum et al., 2011; Matthijs et al., 2016; Sim et al., 2019) and prognostic oncology (Acha et al., 2019; López-Reig et al., 2019). However, stringent regulatory requirements by governing bodies must be met before these technologies can be used for clinical purposes. Hence, their analytical performance must be thoroughly validated.

Research in clinical oncology is constantly expanding the repertoire of clinically-actionable biomarkers such as SNV/Indels, RNA fusions, microsatellite instability (MSI), and tumor mutational burden (TMB), highly complementing pathological insights. Hybridization capture-based NGS assays capable of detecting these biomarkers are commercially available. However, these assays tend to be Caucasian-centric (Park et al., 2018; Yuan et al., 2018), notwithstanding the contribution of genetic diversity, lifestyle (Man et al., 2021; Cheng et al., 2022), and environmental factors (Alexandrov et al., 2016; Lim et al., 2022) to the heterogeneous landscape of cancers.

In this study, an Asian Pan-Cancer Assay (hybridization capture-based) was designed with coding regions from 572 carefully curated Asian-centric cancer biomarkers (Supplementary Table S1) and known recurrent mutations, as well as 91 fusion genes (Supplementary Table S2). The assay’s feasibility, performance, and prospective usage for clinical management were also demonstrated by sequencing more than 200 formalin-fixed, paraffin-embedded (FFPE) tissues across 12 Asian-prevalent cancers with high incidence and mortality in Asia as per Globocan 2020. These include cancers of the stomach, colorectum, breast, lung, biliary tract, liver, cervix, ovary, head and neck, prostate, nasopharynx, and esophagus. Genetic testing performed using this assay can be further integrated into electronic health records, allowing informed treatment strategies and their implementation in conjunction with individualized precision therapy.

## 2 Materials and methods

### 2.1 Sample preparation and extraction

Formalin-fixed paraffin-embedded (FFPE) samples were obtained from the Anatomical Pathology Department of Singapore General Hospital under an Institutional Review Board (IRB)-approved study (CIRB Ref: 2019/2395) following the Human Biomedical Research Act (HBRA). De-identified samples with less than 5 years of archived age were used in this study. For each sample, four unbaked sections of 10-micron thickness with minimally 30% tumor content were used for simultaneous DNA and RNA extraction using Norgen Biotek RNA/DNA FFPE Purification Plus Kit (Cat No. 54300), following the manufacturer’s recommendations. The extracted nucleic acids were subsequently quantified using Qubit dsDNA HS kit (Cat No. Q32851) and Qubit RNA HS Kit (Cat No. Q32855). Nucleic acid was quality controlled using Agilent Genomic DNA TapeStation and Agilent High Sensitivity RNA TapeStation on a TapeStation 4200, following the manufacturer’s protocol.

### 2.2 Probe design, library preparation and sequencing

A probe panel featuring a selection of 572 cancer-related genes and 91 fusion gene candidates was designed based on the Agilent Sure Select Target Enrichment System. During the panel design phase, the extensive literature on high-impact genomic studies of prevalent Asian cancers like liver cancer, bile duct cancer, colorectal cancers with Asian cohort data was referred to enable shortlisting of potential gene candidates. Candidate genes were also shortlisted from genomic data banks like TCGA, ICGC etc. Candidate genes selection focused on those found in Asian only cohorts when a comparison of binary cohorts of Asian vs. others in ICGC and TGCA was performed (Lim et al., 2007; Huang et al., 2016; Jusakul et al., 2017; Ng et al., 2017; Chua et al., 2018; Chan et al., 2020; Zhai et al., 2022). The probe design also included a 16 FEL gene panel that can be used for the complete molecular assessment of fibroepithelial lesions and which has previously been used to characterize a large international cohort of FEL specimens including more than 600 Asian samples (Md Nasir et al., 2019). Candidates with clinical actionability such as those targeted by FDA-approved drugs or used in clinical trials, prognostic in nature, or with specific prevalence in Asians are included in the panel design. An analysis of the overlapping base pairs of this panel vs. a commercially available panel validated by vendors with Caucasian-centric cancers like Melanoma, Sarcoma, and Pancreas cancers also shows low base pairs overlap at less than 40%. The panel covers a 2.33 Mb Region of Interest (ROI) for detecting SNVs, Indels, TMB and MSI and another separate module for detecting RNA-based fusions spanning a ROI of 54.5 kb.

### 2.3 Library construction

The library for each DNA sample was constructed using SureSelect XTHS Target Enrichment System for Illumina Paired-End Multiplexed Sequencing Library, using custom-designed probes described in the earlier paragraph. The kit requires an input of 100 ng of DNA and 40 ng of RNA for their respective library construction, according to the manufacturer’s protocol, unless otherwise stated in this manuscript. After that, an input of 500 ng of indexed libraries prepared from DNA and 200 ng of indexed libraries prepared from RNA was used for target capture, followed by post-capture amplification. The final target-captured libraries were denatured and sequenced with a 5% PhiX spike-in using an Illumina NextSeq 550 system with a paired-end sequencing configuration to achieve a coverage of at least 150X.

### 2.4 Bioinformatics analyses

The bioinformatic analyses for this Asian Pan-Cancer Assay have been integrated into in-house DNA and RNA sequence analysis pipelines, and deployment of the pipelines has been validated. Binary base call (BCL) from data obtained from the NextSeq 550 system was basecalled using bcl2fastq to obtain paired FASTQ sequences. QC was performed on FASTQ sequences before converting the FASTQ sequences to unmapped BAM (uBAM), which embeds metadata information regarding the samples and Unique Molecular Indexes (UMIs).

For detection of SNVs, Indels, TMB and MSI, uBAM was first pre-processed. Sequencing data begins with alignment using bwa (Li and Durbin, 2009) to the GRCh37. p11 genome. These aligned sequences were merged with the initial uBAM to retain metadata and UMI information. Picard MarkDuplicates, part of the GATK4 toolkit (McKenna et al., 2010), was used to flag PCR and optical duplicates in the sequencing library, followed by BaseRecalibration. Variant calling was subsequently performed to identify SNVs and Indels present in the sequenced samples and later annotated using VEP (McLaren et al., 2016) version 95. Potential germline alterations were flagged using population-based annotation such as 1,000 genomes, ExAC and gnomAD databases, included within VEP. SNVs/Indels were prioritized for downstream analysis if the variant was reported within the COSMIC (Bamford et al., 2004) v2 database (accessed from January 2020 to July 2021). TMB was estimated with a scaling factor unique to the panel size while accounting for only coding mutations and Indels. MSI investigation was carried out using in-house developed scripts with in-house determined thresholds.

To detect fusion gene rearrangements, sequencing reads from the fusion module of this panel was pre-processed by mapping sequenced reads to the GRCh37. p11 genome using STAR (Dobin et al., 2013), a splice-aware aligner. A two-pass method was employed during alignment. Fusion calling was performed with ARRIBA (Uhrig et al., 2021). OncoKB (Chakravarty et al., 2017) database (last updated on 29th March 2022 and accessed on 10th April 2022) was used to pull out candidate genomic alterations with actionable targets detected using this assay.

### 2.5 Analytical performance evaluation

#### 2.5.1 Precision

A novel in-house process control formulation containing over 300 verified SNVs was used to assess the precision of SNVs, Indels, MSI and TMB in this assay. Control used also enable precision evaluation of large Indel up to 36 bp, for example, in *MED12*. A total of 20 technical replicates were performed using 30 ng of gDNA input. To assess the precision for fusions, the RNA assay was evaluated using an *ALK-RET-ROS RNA* fusion reference from Horizon Discovery (HD-784) across three individual batches in more than 10 runs with a minimum RNA input of 40 ng.

#### 2.5.2 Linearity for SNV detection

Linearity of the assay was established using Oncospan (Horizon Discovery HD-827) to assess the allele frequency detected for SNV/Indels. The allele frequency for 223 SNVs was compared for Asian Pan-Cancer Assay generated frequency information using three lots of reagents against vendor verified allele frequency by either ddPCR or NGS.

#### 2.5.3 Sensitivity

Sensitivity was evaluated using reference materials with verified SNVs and fusions. For SNV/Indel detection, Oncospan (Horizon discovery, HD-827) was assayed with Asian Pan-Cancer Assay and call concordance for 256 loci overlapping with Asian Pan-Cancer region of interest and have been vendor verified for Oncospan was evaluated with nine replicates. For fusion detection, sensitivity was estimated using five replicates of diluted reference material of moderate RNA quality - Seraseq FFPE Tumor Fusion RNA Ref v4 reference material (catalog number 0710-0496).

#### 2.5.4 Limit of detection

An in-house formulation validated for effectiveness as process control and containing an *NRAS* Q61K mutation with a 5% VAF was used to determine the LoD of this assay. The *NRAS* Q61K at 5% VAF was technically reproduced across five replicates. Other than *NRAS* process control, commercial reference materials of FFPE nature with known allele frequencies determined by the manufacturer using NGS or dd-PCR-based methods were used to confirm the LoD of this assay.

Samples used to access the LoD for fusions were acquired from commercial sources with known and verified fusion copy number information across six technical replicates. Seraseq^®^ FFPE Tumor Fusion RNA Reference Material v2 (catalog number 0710-0129) was used to establish an LoD of 4.65 copies/ng total RNA through serial dilution.

#### 2.5.5 Specificity

Assay specificity for SNV/Indel detection was established using 100 ng Tru-Q 0 (100% Wildtype) Reference Standard (catalogue number HD752, Horizon Discovery) across six technical replicates. This vendor certified reference standard contains four positive SNVs and 40 negative SNVs.

Fusion specificity for the assay was assessed using 5-Fusion Multiplex (Negative Control) across six technical replicates across different manufacturing batches. The reference control was certified by the vendor to be a cell line negative for the fusions: *EML4-ALK*, *CCDC6-RET*, *SLC34A2-ROS1*, *TPM3-NTRK1*, and *ETV6-NTRK3*.

#### 2.5.6 Analytical benchmark—SNV/Indel

10 FFPE samples each with 30 ng of gDNA extracted were used to establish SNV/Indel detection accuracy. As a precaution against degradation of nucleic acid which may confound data, only samples extracted and assayed recently in 2019 and beyond were used for this test. Mixed control (MCO) was also assayed alongside the 10 samples as a form of process control for APC DNA workflow. 100 ng MCO was used. Allele frequency of 40 variants common between the two pan-cancer assays (a commercially available RUO pan-cancer assay vs. APC assay) compared and correlated (Supplementary Table S3).

#### 2.5.7 Analytical benchmark—RNA fusion

The Asian Pan-cancer assay was benchmarked against a commercially available research use only (RUO) assay for fusion detection. Libraries prepared using both assays were sequenced on Nextseq500. 10 FFPE samples were subjected to the RNA workflow for fusion detection. 40 ng of RNA input per sample was used for this benchmark experiment. RNA fusion reference material was assayed alongside the 10 samples as a form of process control for the RNA workflow. The calls made from the two pan-cancer assays were compared to assess concordance among the common fusions targeted by the two panels.

#### 2.5.8 Analytical benchmark—Microsatellite instability

Promega MSI Analysis System, Version 1.2 (catalogue number MD1641), is the current gold standard assay for MSI analysis. The Asian Pan-Cancer DNA workflow validated in this study was benchmarked with the routinely used Promega MSI assay for MSI analysis. Concordance in MSI status was compared between the two methods. Samples tested included reference controls and clinical FFPE samples. A total of 22 FFPE samples and four reference control were analyzed. For Asian Pan-Cancer Assay, 100 ng of DNA extracted from FFPE specimen were used as input per sample. 100 ng DNA extracted from MSI FFPE DNA Reference Standard and 100 ng MSS FFPE DNA Reference Standard (matched MSS control) were used as process control for MSI analysis and included in each batch assayed. For MSI percentage ≥20%, the sample was classified as MSI-H, while the sample was classified as MSS for MSI percentage <20%.

Four nanograms of each sample were used for MSI amplification with Promega MSI Analysis System according to manufacturers instruction, Version 1.2. Similarly, 2 ng of each control was used as input for MSI amplification with Promega MSI Analysis System v1.2 as a form of process control. The fragment analyzer used for this study was Applied Biosystems 3730xl DNA. Five mononucleotide repeat regions were analyzed with the Promega MSI test to determine microsatellite stability status (Supplementary Table S4). The pentanucleotide markers were used to detect potential sample mix-up or contamination. In the presence of mix-up or contamination, the results would be voided and not be used for further analysis. Samples in which ≥40% of microsatellite markers are altered (≥2 altered markers out of 5) are classified as MSI-H. Samples with less than 40% mononucleotide repeat markers altered are classified as MSS.

#### 2.5.9 Analytical benchmark—Tumor mutational burden

Benchmark for TMB estimation by Asian Pan-Cancer Assay was conducted against sample source-verified scores (Supplementary Table S5), which is compared against measurements from vendor/source using whole-genome sequencing (WGS) and/or whole-exome sequencing (WES). Seraseq^®^ FFPE TMB RM material, as listed in Table below, is of 30% tumor content as confirmed by the source. A threshold of 10 mutations/Mb was used as an intermediate TMB score to determine samples with high TMB. A correlation between vendor-supplied TMB scores vs. scores estimated using Asian Pan-Cancer Assay was also performed.

## 3 Results

### 3.1 Panel design

The Asian Pan-Cancer Assay was designed with biomarkers carefully curated from research translations, literature reviews and genomic databases. The hybridization capture-based assay spans a genomic region of interest spanning 2.3 Mb. The assay can identify genetic alterations such as SNVs and Indels, TMB and MSI across 572 protein-coding regions of cancer-relevant genes and their driver mutations (Supplementary Table S1). The assay also features 16 genes spanning a genomic region of 78 kb used to profile fibroepithelial lesions (FELs), distinguishing benign fibroadenomas from potentially malignant phyllodes tumors that are more prevalent in Asian women, thereby complementing histological diagnosis (Tan et al., 2015; Koh et al., 2019; Lim et al., 2019; Md Nasir et al., 2019; Sim et al., 2019; Ng et al., 2021). Of the gene candidates included, 34 candidates are also listed in National Comprehensive Cancer Network (NCCN) guidelines for diagnosis/detection and treatment of breast, colorectal and ovarian cancers (Benson et al., 2018; Carroll et al., 2016; Colevas et al., 2018; Ettinger et al., 2016; Gradishar and Salerno, 2016; Telli et al., 2019). This is as of 14th December 2020 (Supplementary Table S1). An additional 54.5 kb region is used for identifying chimeras across 91 fusion genes during carcinogenesis (Supplementary Table S2).

In this study, the Health Sciences Authority of Singapore and College of American Pathologists (CAP) guidelines were referenced for analytical validation design to ensure the accuracy of reported genetic alterations. The assay was analytically evaluated for precision, the limit of detection (LoD), linearity, sensitivity and specificity using reference material and over 200 clinical FFPE samples for clinical validation (Supplementary Table S6).

### 3.2 Analytical validation

**FIGURE 1:**
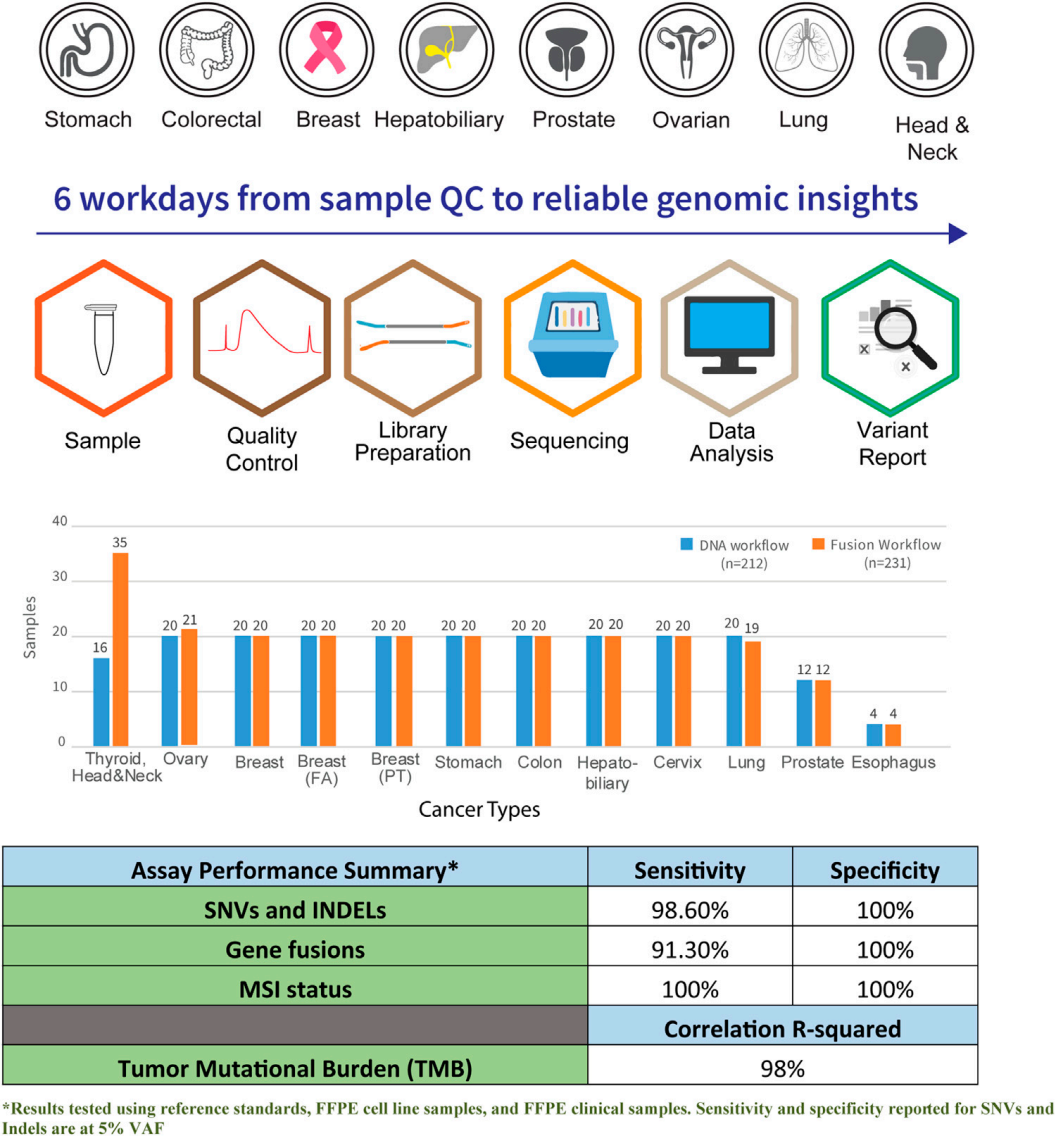
Graphical overview of workflow and coverage of cancer subtypes in the Asian Pan-Cancer Panel. A total of six workdays are required from sample QC to reliable genomic insights. Total number of samples profiled across the various cancer subtypes and assay performance summary are as shown.

The panel’s performance was highly reproducible in a 6-days quality-controlled workflow timeline from sample to clinical insights (Figure 1). Analytical validation was performed to ensure the accuracy of the reported results in this assay. Analytical performance for precision, reportable detection range, specificity, and assay accuracy was established using in-house process control. A reproducibility with 99% concordance from SNVs, Indels, TMB, and MSI analysis was achieved through this validation. The assessment was performed using an in-house process control containing over 300 verified SNVs/Indels across 20 replicates (Supplementary Table S7). Similar analytical performance was obtained for fusion recall using an ALK-RET-ROS reference material across 20 runs. The fusion was confidently recalled across the runs with a concordance of 100%. Identical performance was observed across three different manufacturing lots of this assay.

The reportable range of results for this assay was established through the LoD and linearity of the reported variants. In this study, LoD is the lowest variant allele frequency (VAF) that could be detected in at least 95% of technical replicates under identical input material and quality. The assay was established to have a lower bound target sensitivity of 5% VAF using a gDNA input of 100 ng with process control containing an *NRAS* Q61K mutation and commercial reference materials (Supplementary Table S8). For accurate quantification of VAF, the linearity of the panel was evaluated across three replicates using a total of 223 SNV/Indels using the commercial reference Oncospan (Horizon Discovery HD827) with three different lots of reagents (Supplementary Table S9). For fusions, the LoD was established using the limit of copy number per nanogram of total RNA input, requiring 95% of technical replicates to be accurately recalled. Commercial reference materials with verified fusion information established a lower bound sensitivity of five copies per nanogram for detecting fusions across six technical replicates.

The specificity of this assay was established to understand the likelihood of detecting false positives during SNV/Indel calling. SNV/Indel specificity was confirmed across six technical replicates using a commercial reference standard—Tru-Q 0 (100% Wildtype) Reference Standard (catalogue number HD752, Horizon Discovery) containing four positive mutations and negative for 40 mutations (Supplementary Table S10). 40 variants not present in the parental cell line used to make this standard have been verified to be absent by the vendor. The four positive variants could be used as an internal control to confirm the success of workflow and sequencing, and that variants not picked up ought not to be due to failed workflow but due to absence in the sample used. For fusion detection, specificity was evaluated across three baits lots using 40 ng input of 5-Fusion Multiplex (Negative Control) FFPE section (Horizon Discovery, catalog number HD783) across six technical replicates. This reference material has been verified not to contain *EML4-ALK, CCDC6-RET, SLC34A2-ROS1, TPM3-NTRK1*, and *ETV6-NTRK3* fusions by the vendor, and these fusions should not be detected if the assay is specific. The assay was highly specific for calling SNV/Indel and detecting spliced fusions.

The assay was established to have a median sensitivity of 98% with 100% specificity for genetic aberrations. The analytical performance of the assay was found to be 100% specific, with sensitivity >90% with an LoD at five copies per nanogram of total RNA for fusions. Additionally, the assay showed high linearity and concordance when benchmarked with orthogonal methods such as Promega kit for MSI analysis, whole-genome and exome sequencing for TMB scoring, and RNA fusion transcripts confirmed by endpoint RT-PCR and RNA-seq. The analytical performance of the assay is summarized (Supplementary Table S7).

### 3.3 Clinical validation

**FIGURE 2:**
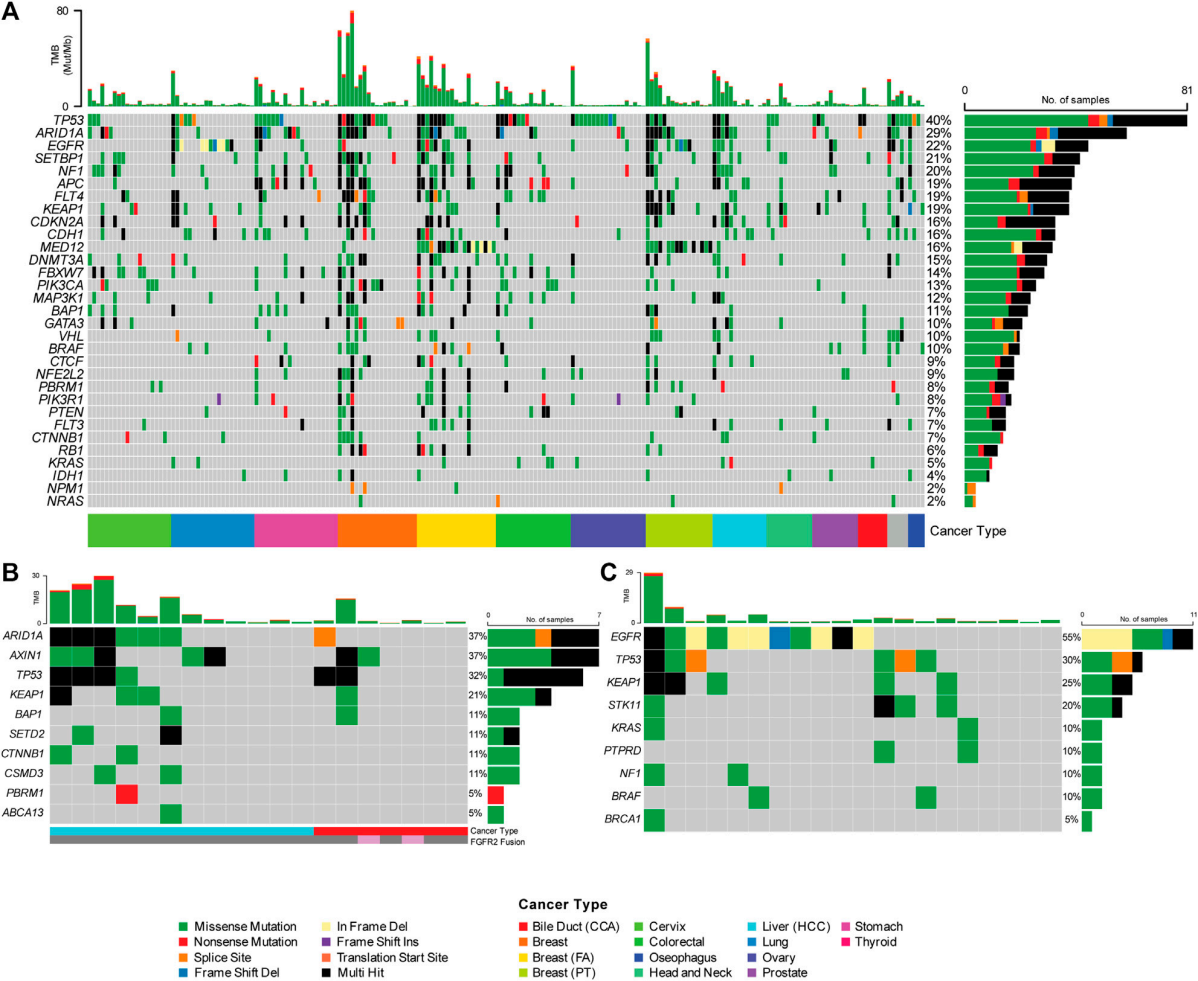
**(A)** Single Nucleotide Variation and Short Indels (SNV/Indels) detected using the Asian Pan-Cancer Assay across 12 varying cancer subtypes. Genetic heterogeneity observed in the Asian cancer samples assayed with Asian Pan-Cancer Assay, as further illustrated with example of the **(B)** Hepatobiliary and **(C)** Lung cancer samples evaluated in this study and their mutation profiles.

A total of 212 FFPE samples across 12 different tumor types were assessed for clinical performance across 17 individual runs (Figure 2; Supplementary Table S11). The FFPE samples ranged between 3−5 years of age. The tumor content of these samples across the cohort profiled for clinical validation was determined to be >60% through pathological assessment. Using this assay, a superior analytical benchmark substantiated with rigorous process controls, confidently identified 64,792 SNVs/Indels across the cohort. Known variants frequently mutated in TGCA and ICGC cohorts are shown in Figure 2A to demonstrate the mutational landscape across the profiled cancers in this cohort. Figure 2B shows common genetic alterations of the hepatobiliary samples profiled as part of clinical validation. This figure shows that the HCC samples harbored increased common alterations in hepatobiliary cancers compared to cancers of the biliary tract. Figure 2C shows genetic alterations from lung samples showing *EGFR* exon 18−20 hotspots and exon 19 frameshift deletions. The identified SNVs/Indels were interrogated over 592 cancer-relevant genes spanning a genomic region of 2.33 Mb.

**FIGURE 3:**
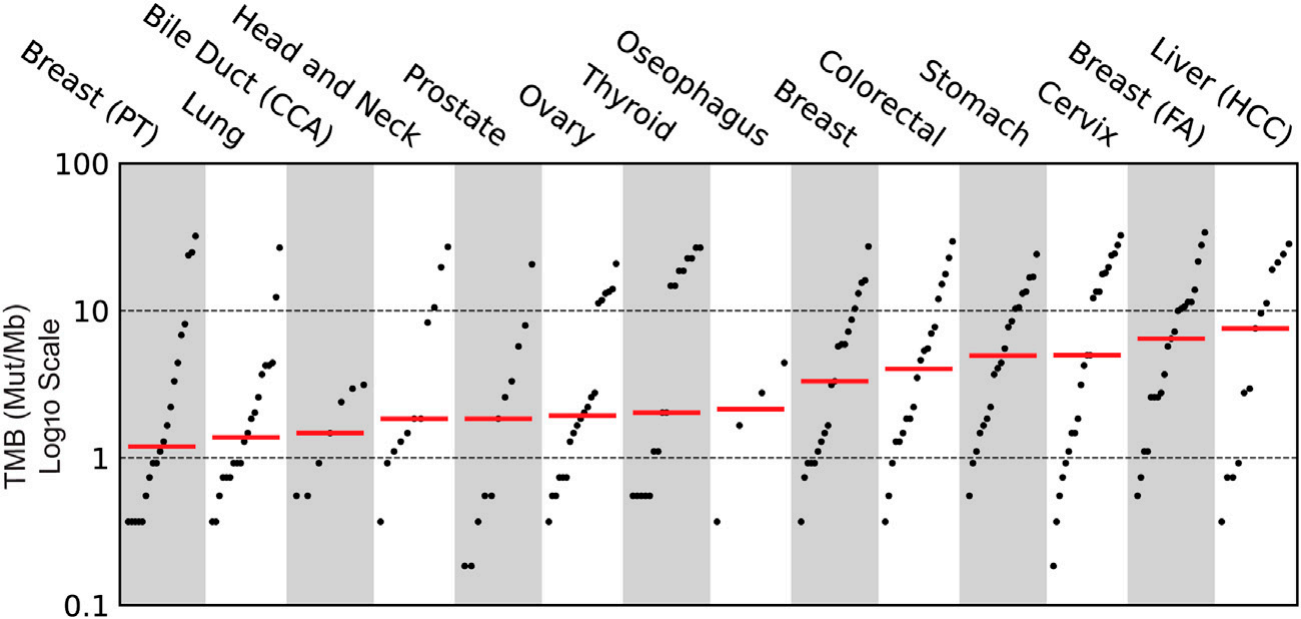
Clinical evaluation of Tumor Mutational Burden (TMB) across the various cancer subtypes profiled using Asian Pan-Cancer Assay.

**FIGURE 4:**
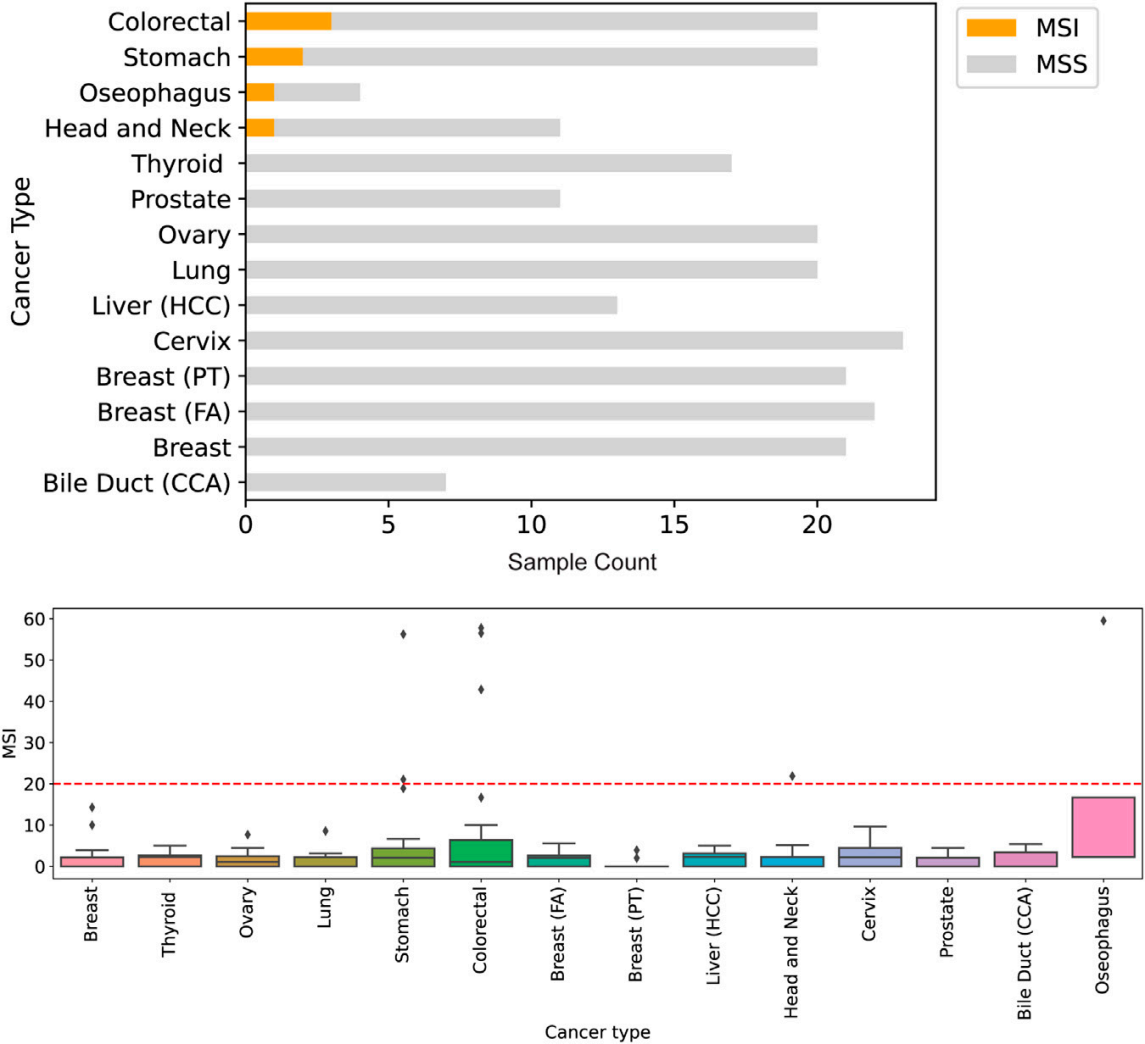
Detection of genomic instability in microsatellite regions of the genome. Across 212 samples profiled, seven were assessed to be microsatelite instable (MSI) by Asian Pan-Cancer Assay and Promega kit for MSI analysis. The highest number of microsatelite instable sites originate tissues of colorectal and gastric cancers.

Clinical evaluation of TMB ranged from 0.4 mutations per megabase (Mut/Mb) to 68.7 Mut/Mb, with a mean TMB of 16.1 Mut/Mb (Figure 3). Seven samples profiled showed the presence of microsatellite instability (Figure 4), including stomach adenocarcinoma (PC148 and PC237), colon adenocarcinoma (PC182, PC105, and PC11), esophagus adenocarcinoma (PC129) and nasopharyngeal carcinoma (PC222). The esophagus adenocarcinoma (PC129) had the highest number of unstable microsatellite sites, with 59.5% of the sites mutated (Supplementary Table S12).

Aside from detecting DNA-based biomarkers, the RNA module of the assay can detect fusion-based gene rearrangements. Across the cohort, six fusion gene rearrangements were identified in cancers of the colon (*TPM-NTRK1*), bile duct (*FGFR2*), thyroid (*BRAF*) and prostate (*ETV4-CLTC*, and *TMPRSS2-ERG*) (Supplementary Table S13). 231 samples were used for validation of the fusion detection workflow.

**FIGURE 5:**
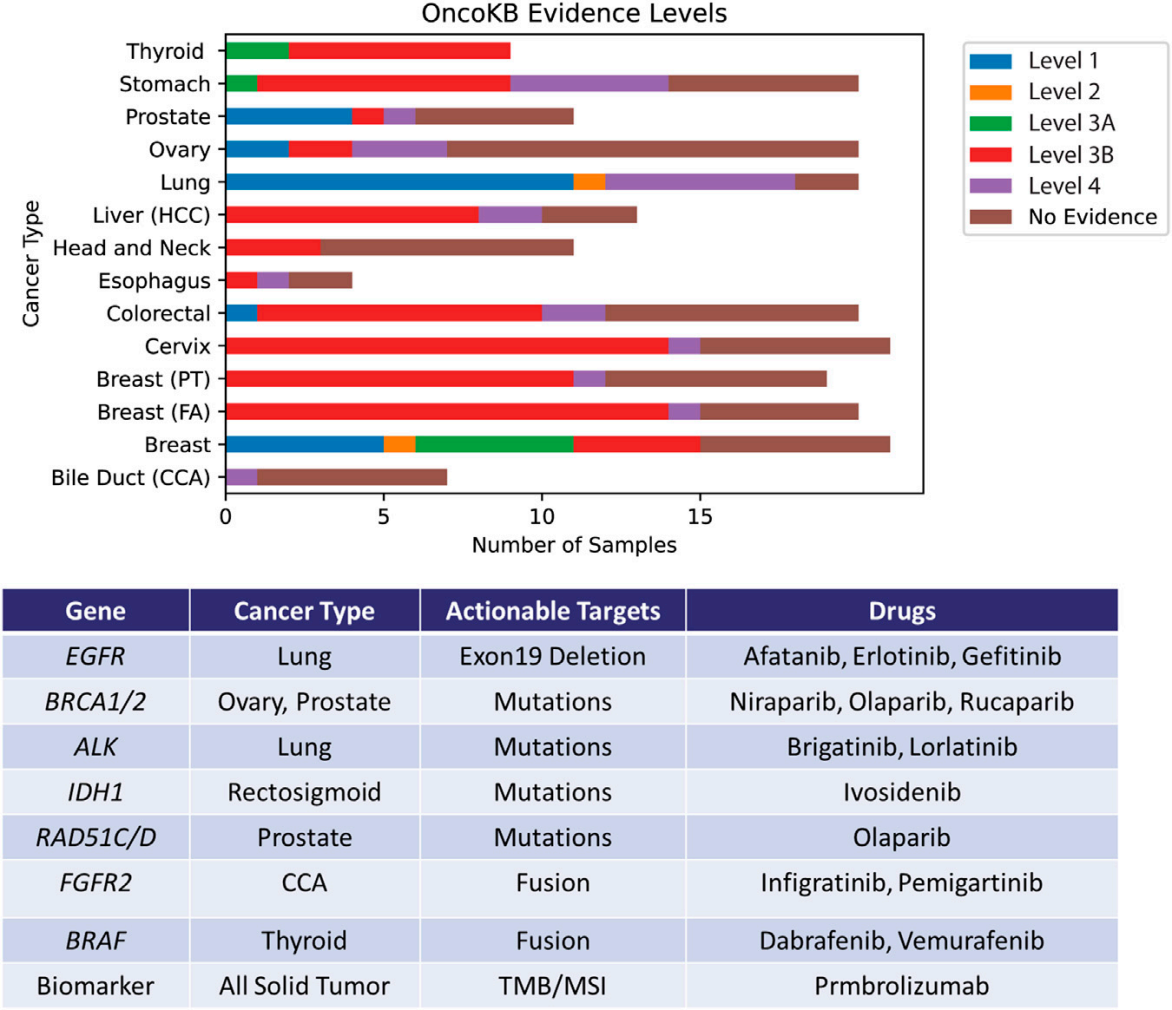
Identification of actionable mutations by the Asian Pan-Cancer Assay by cross-referencing OncoKB database. The levels portrayed depicts varying varying degrees of action ability based on OncoKB evidence across the various cancer subtypes profiled in this study. Examples of potentially applicable drugs matched to the actionable targets are shown in the table.

The identification of actionable targets from DNA and RNA modules of the assay was performed using OncoKB database (Chakravarty et al., 2017). OncoKB has seven varying evidence levels for identifying actionability. In this study cohort, four different levels of OncoKB—1, 2, 3B and 4 were identified. 11% of the cohort harbored at least level 1 actionable evidence, level 2 at 1%, level 3A at 4%, level 3B at 38% and level 4 at 11%. The remaining 35% have no known actionable evidence described yet. In total, 65% of the samples profiled contained at least one mutation with potential actionability (Supplementary Table S14 and Figure 5).

## 4 Discussion

Breakthroughs in cancer research have deepened our biological understanding of tumor progression, evolution and evasion. With efficient treatment regimes, novel treatment strategies targeting major cancer biomarkers have reduced mortality rates. However, validated oncology panels with cancer biomarkers and prevalent cancer types such as bile duct cancers, HCC and clear cell ovarian cancer remains limited, significantly reducing the choice and option of standard diagnostic tests for cancer patients and clinicians. Molecular diagnostic assays with significant cancer biomarkers with recently approved treatment strategies have the potential to offer a wider range targeted therapies for cancer patients, improving survival rates and better quality of life through personalized medicine. An accessible and accurate test can also help to streamline and facilitate timely treatment decision-making. Through this study, an Asian-centric cancer assay was engineered in a LDT setting based on hybrid capture-based sequencing technology and validated with over 200 clinical specimens. The panel features a 16-gene FEL panel used for characterization of fibroepithelial lesions, cancer gene drivers found in Asian cohorts in genomic databases such as TCGA and ICGC. More ongoing studies are currently in progress for other possible Asian-specific or dominant phenotypes to further confirm and expand the utility of this assay as an Asian phenotype prevalence test.

As the deployment of these molecular oncology biomarkers tests plays a vital role in obtaining accurate molecular information for diagnosis and treatment, stringent laboratory requirements must be followed, and thorough validation must be completed. The extensive and rigorous analytical validation conducted in the development of this Asian Pan-Cancer Assay is critical for evaluating and understanding the performance characteristics of the panel. The various characteristic evaluated include assessment of precision, sensitivity, specificity, and analytical accuracy across replicates using analytical reference materials. The results from the analytical validation confirm the assay performance to be robust, reproducible, specific and sufficiently sensitive for an NGS-based broad molecular profiling assay. Before the actual deployment of the assay for clinical purposes, the assay should also be thoroughly evaluated for clinical performance and utility to ensure that its intended use can be fulfilled by the assay.

The ability of the assay to pick up clinically actionable markers or mutations across tumor types is demonstrated by assay performance evaluation with both analytical reference and clinical specimens, giving confidence that this well-validated assay could be helpful for the therapeutic management of patients. This is despite the limitations in sample size and lack of clinical annotations for treatment response and prognosis for the assayed clinical specimens. An example is the ability of this assay to estimate TMB robustly. Clinical studies have noted the association of high TMB with improved patient responses and survival benefits after immune checkpoint inhibitor (ICI) treatment either in a single cancer type (e.g., non-small-cell lung cancer, melanoma, gastric cancer, and urothelial cancer) or in a combined cohort of multiple cancer types. The application of TMB as a biomarker for ICI treatment is now being prospectively tested. Therefore, TMB assessment has become a research hotspot in precision medicine. Currently, whole-exome sequencing (WES) derived TMB values are considered the gold standard, but the high cost and long turnaround time limit the routine diagnostic applicability of WES. Therefore, targeted next-generation sequencing (NGS) panels have been promoted as a more straightforward and cheaper approach for TMB estimation and assessment of microsatellite stability status, potentially aiding clinicians in deciding on immunotherapy blockade treatment approved for genomic instability of TMB and MSI such as the recent FDA approved Keytruda (Pembrolizumab). In 2016, the U. S. Food and Drug Administration granted accelerated approval to Pembrolizumab (KEYTRUDA injection, Merck Sharp & Dohme Corp.) for the treatment of patients with recurrent or metastatic head and neck squamous cell carcinoma (HNSCC) with disease progression on or after platinum-containing chemotherapy. More recently, in June 2020, the Food and Drug Administration approved Pembrolizumab (KEYTRUDA, Merck & Co.) for the first-line treatment of patients with unresectable or metastatic microsatellite instability-high (MSI-H) or mismatch repair deficient (dMMR) colorectal cancer. The validation data from this study shows that the MSI-H samples detected with the MSI analysis pipeline of the Asian Pan-Cancer laboratory-developed test (LDT) are mainly stomach, colon, nasopharynx and esophageal samples. This is coherent with current scientific evidence and indications for use with FDA-approved Keytruda. This confirms that the assay yields clinically relevant data to help guide therapeutic decisions with currently available immune-based therapies.

The treatment outcomes for Asian prevalent malignancies like bile duct cancers are often dismal, and clinicians are advocating for the expansion of the treatment landscape to include more targeted agents and other chemotherapy options through a personalized medicine approach, thus opening doors for various management strategies. For example, FDA has approved orally administered Truseltiq (infigratinib) from Brisbane, California-based QED Therapeutics, Inc., under the accelerated approval program for treating patients with locally advanced or metastatic cholangiocarcinoma (CCA) harboring an *FGFR2* fusion or rearrangement. The same is for another recently approved FDA drug Incyte’s Pemazyre (pemigatinib) which will be Truseltiq’s main competitor for treating CCA harboring *FGFR* mutations. The Asian Pan-Cancer Assay described here with the ability to detect spliced *FGFR2* fusions could be helpful in helping clinicians make decisions regarding prescribing these drugs. Interestingly, this study also noted cholangiocarcinoma (CCA) FFPE clinical specimens with *FGFR2* RNA fusions without the 20 top actionable DNA *NOTCH-1* mutations. On the contrary, the other cholangiocarcinoma samples such as PC47 possessed many of the top 20 genomic mutations such as *NOTCH* mutations but had no *FGFR2* fusion. This reveals possible genetic heterogeneity in the Asian hepatobiliary cancer sample cohort analyzed for this study. The Asian Pan-Cancer Assay could be helpful as a targeted sequencing-based, biomarker-guided treatment selection tool according to individual CCA patient’s biomarkers profiles for best treatment outcomes (Figure 2B).

Aside from detecting RNA fusions, MSI analysis and TMB estimation that could help identify druggable mutations for targeted therapy selection, many actionable SNV/Indel can also be seen with a high sensitivity at a claimed limit detection of 5% VAF using this assay. An example is that of Exon 19 deletion in lung cancer that could inform the use of Tarceva (Erlotinib), a tyrosine kinase inhibitor approved by the U.S. Food and Drug Administration (FDA) for the treatment of certain types of non-small cell lung cancer (NSCLC) that have spread to nearby tissues or other parts of the body. We have also observed similarly actionable SNV/Indel-based mutations with approved drugs in cancer samples aside from lungs, such as that for prostate, breast and ovary.

## 5 Conclusion

The analytical and clinical validation data taken together confirm the usefulness of this assay for the measurement of four major classes of biomarkers - RNA fusions, TMB, SNV/Indel and MSI status, thereby enabling the molecular characterization of Asian prevalent cancers within a short turnaround of 6 days from sample receipt to report generation and issuance. These validation data also demonstrate that the assay can identify genomic variants that may inform therapeutic decisions for cancer patients with a fast turnaround of 6 days from sample QC with minimal carryover. This enables an optimal window for treatment. Through evaluation with FFPE clinical specimens, the clinical validation results provide additional information and data that suggest physicians can use this assay to better characterize Asian prevalent cancers at the molecular level to aid in their clinical decision-making. Prospective consented cohort studies of a larger scale with supplemented clinical and treatment datasets as a follow up study will further confirm the applications of this assay in real-world clinical settings and its clinical utility.

## Data Availability

The data has been deposited to NCBI Sequence Read Archive with Accession number PRJNA851902.

## References

[B1] Acha P. Xandri M. Fuster-Tormo F. Palomo L. Xicoy B. Cabezón M. (2019). Diagnostic and prognostic contribution of targeted NGS in patients with triple-negative myeloproliferative neoplasms. Am. J. Hematol. 94, E264–E267. 10.1002/ajh.25580 31321810

[B2] Alexandrov L. B. Ju Y. S. Haase K. Van Loo P. Martincorena I. Nik-Zainal S. (2016). Mutational signatures associated with tobacco smoking in human cancer. Science 354, 618–622. 10.1126/science.aag0299 27811275 PMC6141049

[B3] Bamford S. Dawson E. Forbes S. Clements J. Pettett R. Dogan A. (2004). The COSMIC (catalogue of somatic mutations in cancer) database and website. Br. J. Cancer 91, 355–358. 10.1038/sj.bjc.6601894 15188009 PMC2409828

[B32] Benson A. B. Venook A. P. Al-Hawary M. M. Cederquist L. Chen Y. J. Ciombor K. K. (2018). NCCN guidelines insights: Colon cancer, Version 2.2018. J. Natl. Compr. Canc. Netw. 16 (4), 359–369. 10.6004/jnccn.2018.0021 29632055 PMC10184502

[B33] Carroll P. R. Parsons J. K. Andriole G. Bahnson R. R. Castle E. P. Catalona W. J. (2016). NCCN guidelines insights: Prostate cancer early detection, Version 2.2016. J. Natl. Compr. Canc. Netw. 14 (5), 509–519. 10.6004/jnccn.2016.0060 27160230 PMC10184498

[B4] Chakravarty D. Gao J. Phillips S. Kundra R. Zhang H. Wang J. (2017). OncoKB: a precision oncology knowledge base. JCO Precis. Oncol. 1, 1–16. 10.1200/PO.17.00011 PMC5586540 28890946

[B5] Chan J. Y. Lim A. H. Boot A. Lee E. Ng C. C.-Y. Lee J. Y. (2020). Whole exome sequencing identifies clinically relevant mutational signatures in resected hepatocellular carcinoma. Liver Cancer Int. 1, 25–35. 10.1002/lci2.14

[B6] Cheng E. Ou F.-S. Ma C. Spiegelman D. Zhang S. Zhou X. (2022). Diet-and lifestyle‐based prediction models to estimate cancer recurrence and death in patients with stage III colon cancer (CALGB 89803/alliance). J. Clin. Oncol. 40, 740–751. 10.1200/JCO.21.01784 34995084 PMC8887946

[B7] Chua B. Tan E. Lim D.-T. Ang M.-K. Tan D. Ng Q.-S. (2018). Real world data on epidermal growth factor receptor (EGFR) tyrosine kinase inhibitors (TKI) use in advanced/metastatic non-small cell lung cancer (NSCLC) with EGFR mutations in Singapore. Ann. Oncol. 29, ix161. 10.1093/annonc/mdy425.033

[B34] Colevas A. D. Yom S. S. Pfister D. G. Spencer S. Adelstein D. Adkins D. (2018). Guidelines insights: Head and neck cancers, Version 1.2018. J. Natl. Compr. Canc. Netw. 16 (5), 479–490. 10.6004/jnccn.2018.0026 29752322

[B8] Dobin A. Davis C. A. Schlesinger F. Drenkow J. Zaleski C. Jha S. (2013). STAR: ultrafast universal RNA-seq aligner. Bioinformatics 29, 15–21. 10.1093/bioinformatics/bts635 23104886 PMC3530905

[B35] Ettinger D. S. Wood D. E. Akerley W. Bazhenova L. A. Borghaei H. Camidge D. R. (2016). NCCN guidelines insights: Non-small cell lung cancer, Version 4.2016. J. Natl. Compr. Canc. Netw. 14 (3), 255–264. 10.6004/jnccn.2016.0031 26957612 PMC10181272

[B36] Gradishar W. Salerno K. E. (2016). NCCN guidelines update: Breast cancer. J. Natl. Compr. Canc. Netw. 14 (5 Suppl.), 641–644. 10.6004/jnccn.2016.0181 27226503

[B9] Huang K. K. Jang K. W. Kim S. Kim H. S. Kim S.-M. Kwon H. J. (2016). Exome sequencing reveals recurrent REV3L mutations in cisplatin-resistant squamous cell carcinoma of head and neck. Sci. Rep. 6, 19552–19610. 10.1038/srep19552 26790612 PMC4726344

[B10] Jusakul A. Cutcutache I. Yong C. H. Lim J. Q. Huang M. N. Padmanabhan N. (2017). Whole-genome and epigenomic landscapes of etiologically distinct subtypes of cholangiocarcinoma. Cancer Discov. 7, 1116–1135. 10.1158/2159-8290.CD-17-0368 28667006 PMC5628134

[B11] Koh V. C. Y. Ng C. C. Y. Bay B. H. Teh B. T. Tan P. H. (2019). The utility of a targeted gene mutation panel in refining the diagnosis of breast phyllodes tumours. Pathol. (Phila.) 51, 531–534. 10.1016/j.pathol.2019.04.005 31272781

[B12] Li H. Durbin R. (2009). Fast and accurate short read alignment with Burrows–Wheeler transform. bioinformatics 25, 1754–1760. 10.1093/bioinformatics/btp324 19451168 PMC2705234

[B13] Lim Y. Iau P. Ali A. Lee S. Wong J. Putti T. (2007). Identification of novel BRCA large genomic rearrangements in Singapore Asian breast and ovarian patients with cancer. Clin. Genet. 71, 331–342. 10.1111/j.1399-0004.2007.00773.x 17470134

[B14] Lim S. Z. Ng C. C. Y. Rajasegaran V. Guan P. Selvarajan S. Thike A. A. (2019). Genomic profile of breast sarcomas: a comparison with malignant phyllodes tumours. Breast Cancer Res. Treat. 174, 365–373. 10.1007/s10549-018-5067-5 30511242

[B15] Lim A. H. Chan J. Y. Yu M.-C. Wu T.-H. Hong J. H. Ng C. C. Y. (2022). Rare occurrence of aristolochic acid mutational signatures in oro-gastrointestinal tract cancers. Cancers 14, 576. 10.3390/cancers14030576 35158844 PMC8833562

[B16] López-Reig R. Fernández-Serra A. Romero I. Zorrero C. Illueca C. García-Casado Z. (2019). Prognostic classification of endometrial cancer using a molecular approach based on a twelve-gene NGS panel. Sci. Rep. 9, 18093–18099. 10.1038/s41598-019-54624-x 31792358 PMC6889294

[B17] Man J. Ni Y. Yang X. Zhang T. Yuan Z. Chen H. (2021). Healthy lifestyle factors, cancer family history, and gastric cancer risk: A population-based case-control study in China. Front. Nutr. 8, 774530. 10.3389/fnut.2021.774530 35004808 PMC8727865

[B18] Matthijs G. Souche E. Alders M. Corveleyn A. Eck S. Feenstra I. (2016). Guidelines for diagnostic next-generation sequencing. Eur. J. Hum. Genet. 24, 1515–5. 10.1038/ejhg.2016.63 PMC5027692 27628564

[B19] McKenna A. Hanna M. Banks E. Sivachenko A. Cibulskis K. Kernytsky A. (2010). The genome analysis toolkit: a MapReduce framework for analyzing next-generation DNA sequencing data. Genome Res. 20, 1297–1303. 10.1101/gr.107524.110 20644199 PMC2928508

[B20] McLaren W. Gil L. Hunt S. E. Riat H. S. Ritchie G. R. Thormann A. (2016). The ensembl variant effect predictor. Genome Biol. 17, 122. 10.1186/s13059-016-0974-4 27268795 PMC4893825

[B21] Md Nasir N. D. Ng C. C. Y. Rajasegaran V. Wong S. F. Liu W. Ng G. X. P. (2019). Genomic characterisation of breast fibroepithelial lesions in an international cohort. J. Pathol. 249, 447–460. 10.1002/path.5333 31411343

[B22] Meldrum C. Doyle M. A. Tothill R. W. (2011). Next-generation sequencing for cancer diagnostics: a practical perspective. Clin. Biochem. Rev. 32, 177–195. 22147957 PMC3219767

[B23] Ng A. W. Poon S. L. Huang M. N. Lim J. Q. Boot A. Yu W. (2017). Aristolochic acids and their derivatives are widely implicated in liver cancers in Taiwan and throughout Asia. Sci. Transl. Med. 9, eaan6446. 10.1126/scitranslmed.aan6446 29046434

[B24] Ng C. C. Y. Md Nasir N. D. Loke B. N. Tay T. K. Y. Thike A. A. Rajasegaran V. (2021). Genetic differences between benign phyllodes tumors and fibroadenomas revealed through targeted next generation sequencing. Mod. Pathol. 34, 1320–1332. 10.1038/s41379-021-00787-w 33727697

[B25] Park S. L. Cheng I. Haiman C. A. (2018). Genome-wide association studies of cancer in diverse populations. Cancer Epidemiol. Biomarkers Prev. 27, 405–417. 10.1158/1055-9965.EPI-17-0169 28637795 PMC5740019

[B26] Sim Y. Ng G. X. P. Ng C. C. Y. Rajasegaran V. Wong S. F. Liu W. (2019). A novel genomic panel as an adjunctive diagnostic tool for the characterization and profiling of breast Fibroepithelial lesions. BMC Med. Genomics 12, 142–214. 10.1186/s12920-019-0588-2 31647027 PMC6813086

[B27] Sung H. Ferlay J. Siegel R. L. Laversanne M. Soerjomataram I. Jemal A. (2021). Global cancer statistics 2020: GLOBOCAN estimates of incidence and mortality worldwide for 36 cancers in 185 countries. CA. Cancer J. Clin. 71, 209–249. 10.3322/caac.21660 33538338

[B28] Tan J. Ong C. K. Lim W. K. Ng C. C. Y. Thike A. A. Ng L. M. (2015). Genomic landscapes of breast fibroepithelial tumors. Nat. Genet. 47, 1341–1345. 10.1038/ng.3409 26437033

[B37] Telli M. L. Gradishar W. J. Ward J. H. (2019). NCCN guidelines updates: Breast cancer. J. Natl. Compr. Canc. Netw. 17 (5.5), 552–555. 10.6004/jnccn.2019.5006 31117035

[B29] Uhrig S. Ellermann J. Walther T. Burkhardt P. Fröhlich M. Hutter B. (2021). Accurate and efficient detection of gene fusions from RNA sequencing data. Genome Res. 31, 448–460. 10.1101/gr.257246.119 33441414 PMC7919457

[B30] Yuan J. Hu Z. Mahal B. A. Zhao S. D. Kensler K. H. Pi J. (2018). Integrated analysis of genetic ancestry and genomic alterations across cancers. Cancer Cell 34, 549–560. 10.1016/j.ccell.2018.08.019 30300578 PMC6348897

[B31] Zhai W. Lai H. Kaya N. A. Chen J. Yang H. Lu B. (2022). Dynamic phenotypic heterogeneity and the evolution of multiple RNA subtypes in hepatocellular carcinoma: the PLANET study. Natl. Sci. Rev. 9, nwab192. 10.1093/nsr/nwab192 35382356 PMC8973408

